# The multifunctional protein YB-1 potentiates PARP1 activity and decreases the efficiency of PARP1 inhibitors

**DOI:** 10.18632/oncotarget.25158

**Published:** 2018-05-04

**Authors:** Elizaveta E. Alemasova, Konstantin N. Naumenko, Tatyana A. Kurgina, Rashid O. Anarbaev, Olga I. Lavrik

**Affiliations:** ^1^ Institute of Chemical Biology and Fundamental Medicine, Siberian Branch of Russian Academy of Sciences (SB RAS), Novosibirsk, 630090, Russia; ^2^ Novosibirsk State University, Novosibirsk, 630090, Russia

**Keywords:** Y-box binding protein 1 (YB-1), poly(ADP-ribose) polymerase 1 (PARP1), poly(ADP-ribose) (PAR), PARP1 inhibitors, olaparib

## Abstract

Y-box-binding protein 1 (YB-1) is a multifunctional cellular factor overexpressed in tumors resistant to chemotherapy. An intrinsically disordered structure together with a high positive charge peculiar to YB-1 allows this protein to function in almost all cellular events related to nucleic acids including RNA, DNA and poly(ADP-ribose) (PAR). In the present study we show that YB-1 acts as a potent poly(ADP-ribose) polymerase 1 (PARP1) cofactor that can reduce the efficiency of PARP1 inhibitors. Similarly to that of histones or polyamines, stimulatory effect of YB-1 on the activity of PARP1 was significantly higher than the activator potential of Mg^2+^ and was independent of the presence of EDTA. The C-terminal domain of YB-1 proved to be indispensable for PARP1 stimulation. We also found that functional interactions of YB-1 and PARP1 can be mediated and regulated by poly(ADP-ribose).

## INTRODUCTION

The long-lasting investigation of principles underlying DNA repair and its regulation has drawn the attention of researchers as a basis for the development of new approaches for cancer therapy. Most up-to-date treatment strategies imply agents inducing DNA damage in tumor cells. The significant increase in efficiency is reached by a combination of chemotherapy and drugs inhibiting DNA repair enzymes [1]. One of the most promising targets in this respect is poly(ADP-ribose) polymerase 1 (PARP1), the key regulator of DNA repair events [2].

PARP1 transition from an inactive to active state occurs upon interaction with exposed bases at the site of DNA damage believed to induce restructuring of the protein auto-inhibitory domain [3]. Activated PARP1 synthesizes long (about 200–300 monomers) and branched chains of poly(ADP-ribose) (PAR) using NAD+ as a substrate [4]. The functions of PAR in DNA repair are extremely numerous. This polymer represents a unique molecule as it combines features of *posttranslational modification* modulating protein functions and localization and of *nucleic acid* recognized by DNA-, RNA- as well as specific PAR-binding protein modules [5]. Moreover, PARP1 had been recently shown to modify strand break termini suggesting the possible role of poly(ADP-ribose) in bridging broken DNA molecules similar to the role supposed for small non-coding RNAs [6–8]. Finally, Altmeyer and co-authors demonstrated that PAR nucleates non-membranous compartmentalization at sites of DNA damage [8].

Five PARP inhibitors (PARPi) are now being investigated in randomized, phase III clinical trials [9]. The most extensively studied one, olaparib, was the first PARP1i approved by the Food and Drug Administration (FDA) and European Medicines Agency (EMA) for use as a maintenance monotherapy specifically in patients with germline *BRCA1* or *BRCA2* gene mutations [10]. Despite PARPi hold great promise, either as single agents in the treatment of cancers with defective homologous recombination mechanisms or in combination with chemo- and radiotherapy in a wider spectrum of malignancies, increasing evidence indicates the appearance of resistance to these drugs [2]. The important clinical mechanism of this resistance based on numerous observations is the restoration of functional homologous recombination (HR) in the tumor cells due to secondary mutations in *BRCA1/2* or other core HR pathway genes under PARPi selection pressure [10–13]. Additional mechanisms proposed include elevated expression of transmembrane transporters, such as the *Multidrug resistance* protein (MDR1), reduced activity of the nonhomologous end-joining (NHEJ) factor 53BP1, stabilization of mutant BRCA1 protein by HSP90 [14] or alteration in PARP1 protein levels [15]. The discovery of the molecular mechanisms underlying resistance of tumors to DNA-damaging drugs, including PARPi, and identification of potential biomarkers, intrinsic to resistant cells, is highly topical nowadays.

Two decades ago, overexpression of the Y-box-binding protein 1 (YB-1)/its nuclear localization were found to be associated with tumor phenotype [16]. The changes of YB-1 expression/localization profile reached a maximum in advanced and aggressive tumors resistant to chemotherapy [17]. According to the large body of data assembled, YB-1 is able to desensitize cancer cells (including cancer stem cells) to different kinds of drugs thus significantly reducing the possibility of non-relapsive recovery [18–24]. In this regard, YB-1 may contribute to drug efflux mechanisms, as its overexpression/nuclear localization were found to correlate with activation of the *MDR1* gene [25–27]. Alternatively, taking into account the YB-1 stress-induced nuclear localization [28], increased affinity for damaged DNA and multiple physical and functional interactions with DNA repair factors (reviewed in [29]), a potential role of YB-1 in regulation of DNA repair may also be proposed. Interestingly, this protein has been recently identified as a target of poly(ADP-ribosyl)ation [30] and shown to physically interact with PARP1 as well as to modulate its catalytic activity depending on the level of DNA damage [31].

In the present study, we have applied the real-time technique to explore YB-1-PARP1 interplay during the poly(ADP-ribosyl)ation process. Here we report for the first time the ability of YB-1 to interfere with the action of PARP1 inhibitors. We also show that YB-1 can stimulate PARP1 in the absence of magnesium, and that YB-1-PARP1 interplay can be mediated and regulated not only by the DNA-cofactor at the initial stage of poly(ADP-ribosyl)ation [31], but also by poly(ADP-ribose) during elongation.

## RESULTS

### YB-1 and PARP1 can form a heteromeric complex with damaged DNA

It was shown earlier by fluorescence titration technique that YB-1 can physically interact with PARP1, and this interaction is not disrupted in the presence of damaged DNA [31]. According to these data, PARP1 binding to YB-1 or to the YB-1-DNA complex could be followed by the increase in fluorescence intensity of labelled YB-1 molecule carrying a fluorophore [31].

**Figure 1:**
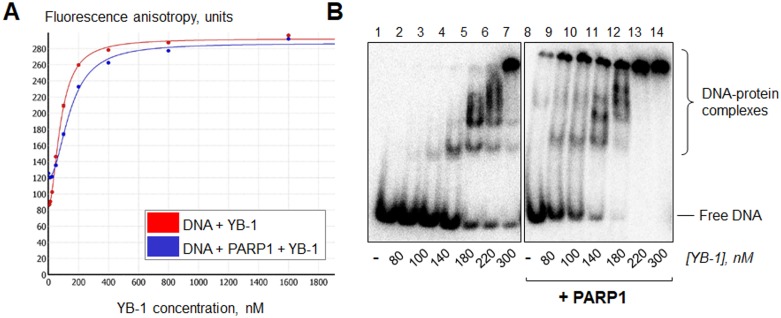
YB-1 and PARP1 are able to form a heteromeric complex with damaged DNA (**A**) The reaction mixtures contained 1× RB, 0 (red curve) or 200 nM (blue curve) PARP1, 100 nM FAM-labelled DNA Nick and 0–1600 nM YB-1. The formation of YB-1-PARP1-DNA complexes was followed by the fluorescence spectroscopy technique. (**B**) Reaction mixtures contained 1× RB, 0 or 200 nM PARP1, 100 nM radioactively labelled DNA Nick and 0–400 nM YB-1. The formation of YB-1-PARP1-DNA complexes was analyzed by gel-shift and autoradiography as described in Materials and Methods. Lanes 1–7: YB-1 binding to DNA in the absence of PARP1; lanes 8–14: in the presence of 200 nM PARP1. The concentrations of YB-1 in the mixtures are presented at the bottom of the panel. Positions of DNA and its complexes were visualized by phosphorimaging; the data acquired were analyzed by the Quantity One analysis software, providing the Transform and Crop Plot tools to optimize the image display. The experiment was performed 2 times.

**Figure 2:**
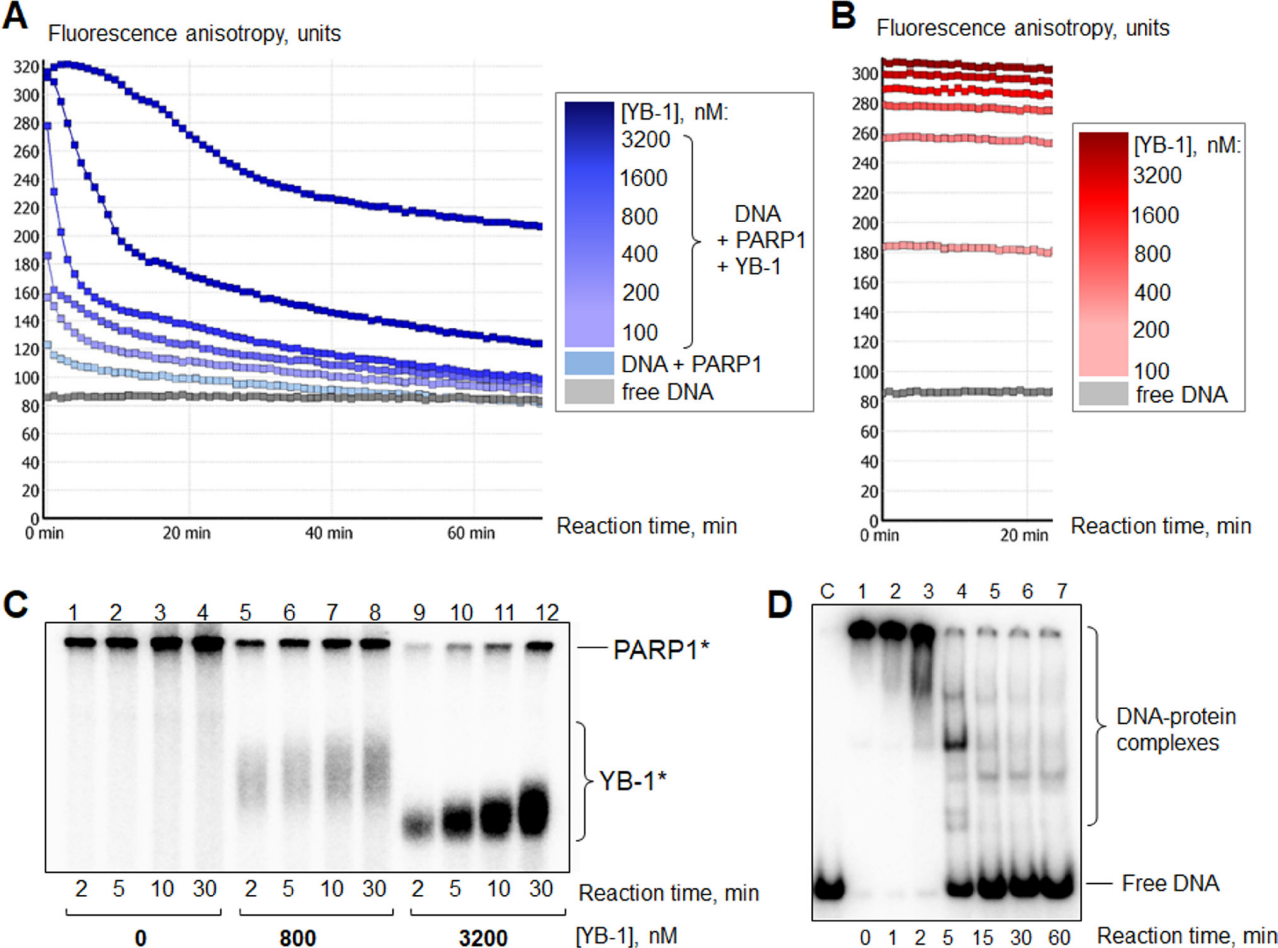
YB-1 is a preferable target of poly(ADP-ribosyl)ation (**A**) The “lag-period” for DNA release at high YB-1 concentration. The curves presented illustrate fluorescence anisotropy change of FAM-labelled DNA Nick measured by kinetic scanning. The reaction mixtures contained 1× RB, 200 nM PARP1, 100 nM Nick and 0–3200 nM YB-1 (the increase in YB-1 concentration is shown by the increased intensity of the color of the curve). Poly(ADP-ribosyl)ation was started by the addition of NAD+ at 25 s to a final concentration of 500 μM. All the measurements were carried out in duplicates for each reaction mixture. (**B**) The presence of PARP1 in the complex is necessary for DNA release after NAD+ addition (control). The curves presented illustrate the change of fluorescence anisotropy of FAM-labelled DNA Nick measured by kinetic scanning. The reaction mixtures contained 1× RB, 100 nM Nick and 0–3200 nM YB-1 (the increase in YB-1 concentration is shown by the increased intensity of the color of the curve). NAD+ was added at 25 s to a final concentration equal to 500 μM. All the measurements were carried out in duplicates for each reaction mixture. (**C**) PARP1 autopoly(ADP-ribosyl)ation reaction performed with the use of radioactively labeled NAD+^*^. Lanes (1–4): without YB-1; (5–8): in the presence of 800 nM YB-1; (9–12): in the presence of 3200 nM YB-1. PARP1^*^, YB-1^*^ designate poly(ADP-ribosyl)ated PARP1 and YB-1, respectively. Reaction times and YB-1 concentrations are shown at the bottom of the panel. Positions of protein bands were visualized by phosphorimaging; the data acquired were analyzed by the Quantity One analysis software, providing the Transform and Crop Plot tools to optimize the image display. The experiment was performed 3 times. (**D**) Gel-mobility shift assay analysis of YB-1 interaction with radioactively labelled DNA Nick (40 nM) during the time of poly(ADP-ribosyl)ation. C1: control for Nick. Lane 1: Nick bound by 400 nM YB-1 and 100 nM PARP1 before NAD+ addition. Lanes 2–7: appearance of free Nick during YB-1 and PARP1 repulsion from the protein-DNA complex upon poly(ADP-ribosyl)ation. The reaction time is shown at the bottom of the panel. The positions of DNA and DNA-protein complexes were visualized by phosphorimaging; the data acquired were analyzed by the Quantity One analysis software, providing the Transform and Crop Plot tools to optimize the image display. The experiment was performed at least 3 times.

To confirm the ability of YB-1 to associate with the PARP1-DNA complex, the fluorescence spectroscopy and gel-shift analysis techniques were used (Figure 1). By fluorescence spectroscopy, we observed YB-1 binding to DNA (Figure 1A, red curve) or to the PARP1-DNA complex (Figure 1A, blue curve). In this case, the formation of a hypothetical ternary complex YB-1-PARP1-DNA could be detected by increase of the fluorescence anisotropy level during YB-1 addition to DNA bound by PARP1 (Figure 1A, blue curve). The presence of PARP1 in this complex could be further confirmed by the PARylation reaction, induced by NAD+ addition (Figure 2A and 2B). By gel-shift analysis we observed that PARP1 stimulated YB-1 binding to radioactively labelled DNA (Figure 1B, compare lanes 1–7 and 8–14), resulting in the formation of DNA-protein assemblies with low mobility in gel, presumably corresponding to YB-1-PARP1-DNA complexes (Figure 1B, lanes 10–13).

The question remains as to whether PARP1 auto-poly(ADP-ribosyl)ation occurs within PARP1 dimers or not [32]; however, several proteins were shown to regulate PARP1 activity [33–38]. It may be proposed that the formation and stoichiometry of the heteromeric complex of PARP1 and its partner protein on DNA damage are significant for the regulation of PARP1 activity. However, the initial formation of this complex appears to be especially important for PAR-binding proteins, as high concentrations of poly(ADP-ribose) generated after PARP1 activation may disconnect functional coupling of the partners.

### In the heteromeric complex with PARP1 and DNA, YB-1 is a preferable PAR acceptor

**Figure 3:**
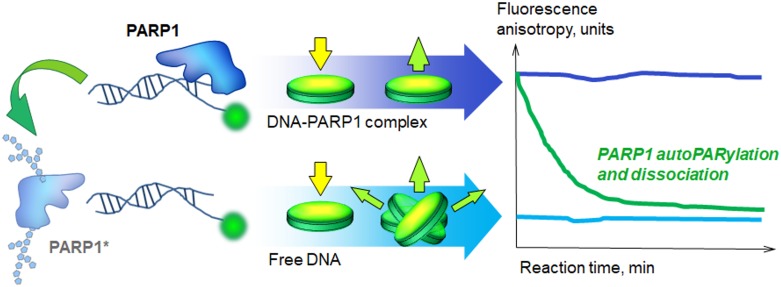
Real-time assay for poly(ADP-ribosyl)ation Reaction mixtures were prepared in Corning black 384-well polystyrene assay plates and irradiated with polarized light. Fluorescence anisotropy was defined as the ratio of the polarized component to the total intensity: A = (I_1_ – I_2_) / (I_1_ + 2I_2_), where I_1_ and I_2_ are the intensities of the light emitted by a fluorophore along different axes of polarization. The anisotropy level was used to estimate the size of the complex containing fluorescent DNA. During irradiation, excitation of the fluorophore can occur only if the electric field of the light is oriented in a particular axis towards the molecule. The anisotropy value (A) is maximum when the rotation of the fluorophore is confined by proteins bound to fluorescently labelled DNA and I_1_ >> I_2_ (that correspond to the case of unmodified PARP1, purple curve). The minimum A level is observed when the fluorophore has high mobility and I_1_ ~ I_2_ (that in our conditions corresponds to the control sample containing only DNA (blue curve) or free DNA after repulsion of poly(ADP-ribosyl)ated PARP1 (green curve)). The method allows one to detect protein binding to / dissociation from fluorescent DNA in the real-time.

Recently YB-1 was identified as a target of poly(ADP-ribosyl)ation [30]. However, YB-1 and PARP1 interplay on damaged DNA has to date not been studied in real time. Fluorescence spectroscopy assay is the only technique applicable for real-time detection of the poly(ADP-ribosyl)ation existing to date, because other methods for PARP1-catalyzed reaction are based on the estimation of the amount of unreacted NAD+ or poly(ADP-ribose) generated [39, 40]. By fluorescence spectroscopy, PARylation process could be detected indirectly by a change in fluorescence anisotropy of the FAM-labelled DNA-cofactor, as poly(ADP-ribosyl)ation of PARP1 results in its dissociation from the complex with damaged DNA due to electrostatic repulsion between negatively charged DNA and the growing polymer of poly(ADP-ribose) [41] (Figure 3).

By using this method, we could follow the formation of DNA-protein complexes (YB-1 and PARP1 can form a heteromeric complex with damaged DNA, Figure 1A, Figure 2A and 2B, at 0 min) and DNA release after NAD+ addition due to dissociation of PARylated YB-1 and PARP1 (Figure 2A, blue curves). As a control, we reproduced the same experiments with the mixtures containing DNA, NAD+ and YB-1 at different concentrations, but no PARP1 (Figure 2B). In fact, we detected no change of fluorescent anisotropy values with the duration of the reaction (Figure 2B, compare with Figure 2A).

The initial inhibition of PARP1 activity (decreased modification of both PARP1 and YB-1) at high [YB-1]:[DNA] ratio ([YB-1] >> [DNA]) was shown by us previously [31]. Here we confirmed that high concentrations of YB-1 in the mixture cause a “lag-period” of DNA release (at ~0–5 min) (Figure 2A, the darkest curve). It can also be observed by gel-shift analysis of the poly(ADP-ribosyl)ation time course with the use of radioactively labelled DNA and unlabelled NAD+ (Figure 2D, lanes 1–3). However, the fall in the level of fluorescence anisotropy (Figure 2A, after 5 min) due to accumulation of free DNA (Figure 2D, lanes 4–7) observed in the course of time is evidence of an active poly(ADP-ribosyl)ation process. These data speak in favor of the fact that PARP1 is not inhibited. We suppose that this phenomenon can be accounted for the preferred poly(ADP-ribosyl)ation of YB-1 molecules accompanied by a slowdown of PARP1 auto-modification. In this case, dissociation of DNA-protein complexes is retarded by binding of new YB-1 molecules, replacing modified YB-1 molecules that have low affinity for DNA [30]. This process of exchange may continue until the unmodified YB-1 pool would be depleted. To confirm this hypothesis, we reproduced the experiment with the use of radioactively labelled NAD+^*^ to detect the reaction products (Figure 2C). Indeed, in the reaction conditions used, YB-1 was the main target of modification, while the level of PARP1 auto-modification was slightly decreased (Figure 2C, compare lanes 1–4 and 9–12). In this regard, not only YB-1 and PARP1 competition for DNA as was proposed earlier [31], but also their competition for poly(ADP-ribose) appears to contribute to YB-1-mediated inhibition of PARP1 auto-modification, observed at the early stages of the poly(ADP-ribosyl)ation reaction at a high [YB-1]:[DNA] ratio ([YB-1] >> [DNA]).

### YB-1 stimulates PARP1 activity in the absence of magnesium

**Figure 4:**
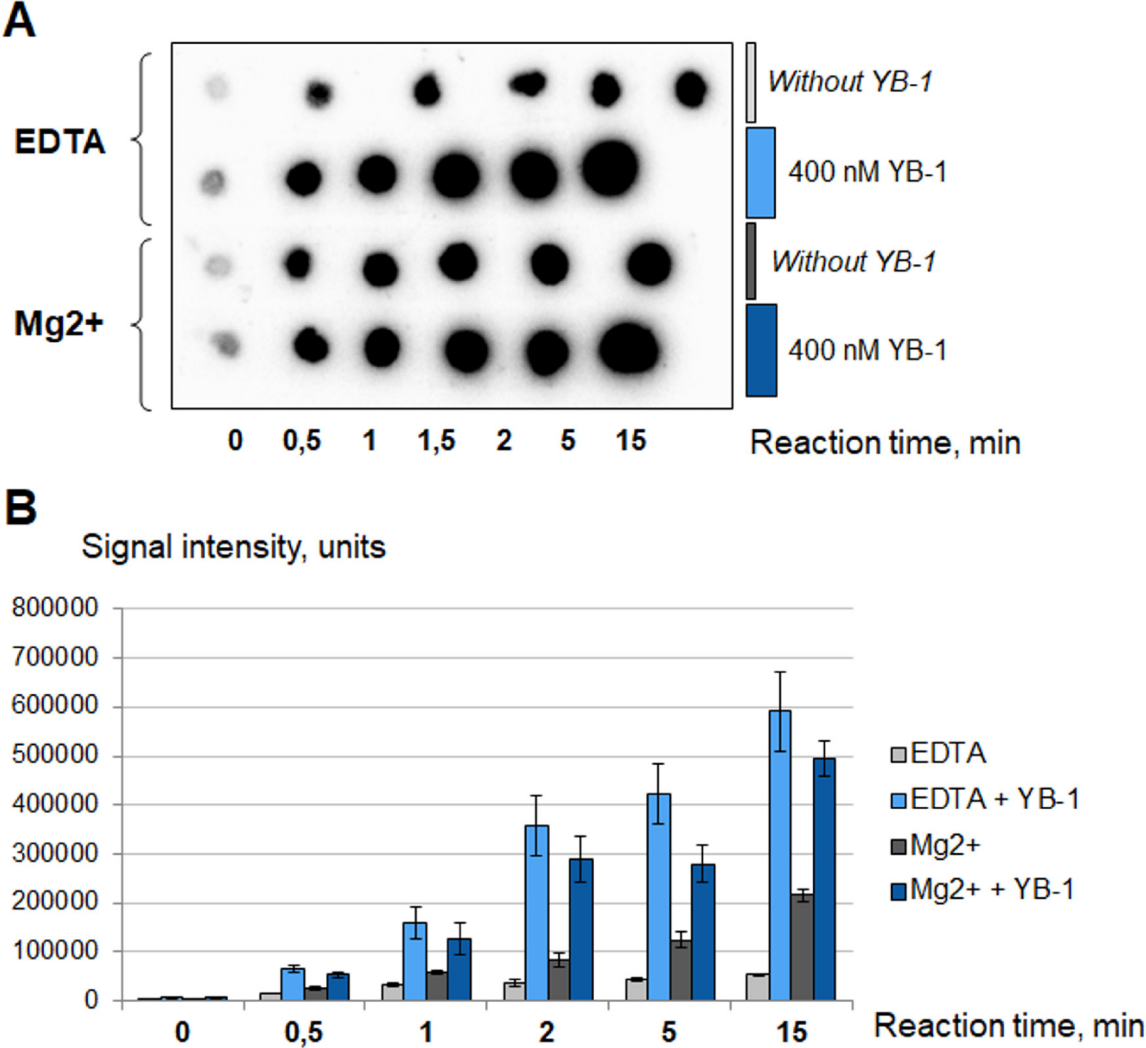
YB-1 stimulates PARP1 activity in the absence of magnesium The poly(ADP-ribosyl)ation reaction was performed using radioactively labelled NAD+^*^ as described in Materials and Methods and analyzed using TCA-targets. The radioautographs of TCA-targets optimized by Quantity One Transform Plot tool (**A**) are presented. The reaction buffer contained 5 mM MgCl_2_ or 10 mM EDTA. Reactions were performed without YB-1 (gray columns and the first series of TCA-targets) or in the presence of 400 nM YB-1 (blue columns and the second series of TCA-targets). The reaction time is shown at the bottom of the panel A. The experiment was performed at least three times, the histogram (**B**) shows the mean values ± SD of three independent experiments.

It was found previously that damaged DNA itself can not serve as effective cofactor for PARP1 in the absence of cations [42]. The addition of EDTA to the reaction mixture abrogates PARP1 activation by Mg^2+^ or Ca^2+^ [42], in accordance with the results of our experiments (Figure 4, compare PARP1 activity in the presence of 5 mM Mg^2+^ or 10 mM EDTA).

Polyamines and histones act as PARP1 cofactors, with the latter possessing activator potential about three order of magnitude higher than bivalent cations [42]. Both polyamines and histones can function regardless of the presence of EDTA; interestingly, bivalent cations in moderate amounts display a synergistic action [42].

**Figure 5:**
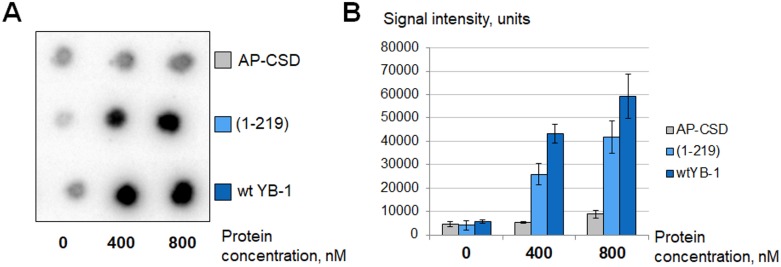
C-terminal domain of YB-1 is necessary for PARP1 stimulation Poly(ADP-ribosyl)ation reaction performed using radioactively labelled NAD+^*^ as described in Materials and Methods and analyzed using TCA-targets. The figure illustrates the radioautographs of TCA-targets (**A**) and the histogram showing the mean values ± SD of three independent experiments (**B**). The reaction buffer was without MgCl_2_ and additionally supplemented with 10 mM EDTA. Reactions were performed in the presence of YB-1 or its mutants in varying concentrations as shown at the bottom of the panels.

In accordance with our results, YB-1 is a significantly more effective PARP1 activator than Mg^2+^. This protein can stimulate PARP1 activity in the presence of 10 mM EDTA as well as in the presence of 5 mM Mg^2+^ (Figure 4). The hallmark of YB-1 is its unusually high isoelectric point (pI(YB-1) = 9.87; calculated from the YB-1 sequence with the ExPASy ProtParam tool). The YB-1 positive charge is located in the C-terminal domain (CTD) of the protein as can be seen from the isoelectric points calculated for the YB-1 nuclear form (pI(YB-1(1-219)) = 9.84) and mutant lacking CTD (pI(AP-CSD) = 6.58; ExPASy ProtParam tool). By using YB-1(1-219) and AP-CSD we confirmed that CTD of YB-1 is indeed responsible for the ability of YB-1 to stimulate PARP1 (Figure 5).

It should be noted that YB-1 as well as Mg^2+^ is unable to stimulate PARP1 activity in the absence of damaged DNA (Supplementary Figure 1). The preparation of YB-1, in which the YB-1 protein was degraded by proteinase K treatment, is also unable to stimulate the activity of PARP1 (Supplementary Figure 2).

### YB-1 interferes with low doses of PARP1 inhibitors, but is unable to restore PARylation by inhibited PARP1

Competitive inhibitors of NAD+ binding with PARP1 such as 3-aminobenzamide were historically the first inhibitors of PARP1. However, the relatively high half maximal inhibitory concentration (IC_50_) of 3-aminobenzamide makes this inhibitor poorly applicable for clinical use [43]. At the present time olaparib and its analogs are the most promising drugs in anticancer therapy. The mechanism of action of olaparib has long been considered as complex, including the competitive inhibition of PARP1 activity and impeding PARP1 dissociation from damaged DNA (*allosteric PARP1 trapping*) that blocks initiation of DNA repair [44]. However, recent studies indicated that trapping is due to catalytic inhibition and not to allosteric PARP1 trapping [45]. *Minor groove binding ligands (MGBLs)* or small DNA-binding molecules such as EtBr can also be used as PARP1 inhibitors, because these agents prevent activation of PARP1 by disturbing its binding to DNA [46].

**Figure 6:**
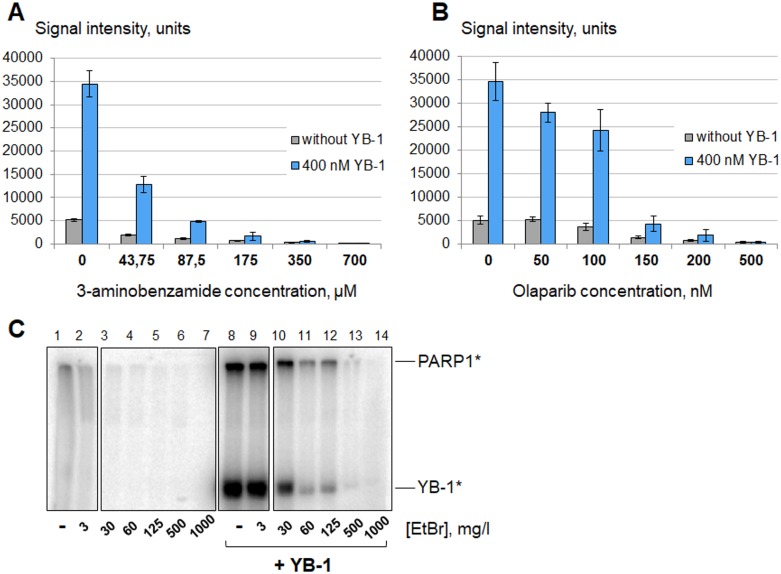
YB-1 stimulates PARP1 activity in the presence of PARP1 inhibitors Poly(ADP-ribosyl)ation reactions performed using radioactively labelled NAD+^*^ as described in the presence of 3-aminobenzamide (**A**), olaparib (**B**) or EtBr (**C**) at varying concentrations and analyzed using TCA-targets or by SDS-PAGE. (A and B) The figures present the histograms obtained by analysis of the radioautographs of TCA-targets by Quantity One software. The reactions were performed in the absence of magnesium (10 mM EDTA) without YB-1 (gray columns) or in the presence of 400 nM YB-1 and 10 mM EDTA (blue columns). The relative PARP1 activity is indicated on the left of the histograms, and the concentrations of inhibitors used are shown at the bottom of the panels. Histograms A and B show the mean values ± SD of three independent experiments. (C). The radioautograph of the SDS-PAGE used to analyze the reaction products. Reactions were performed in the absence of magnesium (10 mM EDTA) without YB-1 (lanes 1–7) or in the presence of 400 nM YB-1 and 10 mM EDTA (lanes 8–14). The concentrations of EtBr are shown at the bottom of the panels. The experiment was reproduced at least three times.

We have shown that the ability of YB-1 to stimulate PARP1 allows this protein to sustain a relatively high level of PARylation in the presence of low concentrations of different PARP1 inhibitors – competitive (0–87.5 μM 3-aminobenzamide, Figure 6A and 0–150 nM olaparib, Figure 6B) as well as small DNA-binding molecules (0–125 mg/l EtBr, Figure 6C). Apparently this phenomenon is due to YB-1-mediated stimulation of PARP1 molecules that avoided inactivation by inhibitors, as YB-1 is unable to enhance PARylation in the presence of high concentrations of inhibitory agents (≥ 175 μM 3-aminobenzamide, Figure 6A; ≥ 200 nM olaparib, Figure 6B; ≥ 500 mg/l EtBr, Figure 6C).

### Stimulation of PARP1 auto-poly(ADP-ribosyl)ation by YB-1 is partially PAR-mediated

Previously it was proposed that YB-1 binding to PAR polymers attached to PARP1 may screen the negative charge of poly(ADP-ribose) thus prolonging PARP1 location on the damaged DNA and the active state of the enzyme [31]. It is possible that even short poly(ADP-ribose) chains attached to PARP1 are sufficient for its repulsion from the catalytically active complex with damaged DNA, while positively charged molecules (Ca^2+^, Mg^2+^, polyamines or histones [42]) or DNA- and PAR-binding proteins (such as XPA [38]) allow poly(ADP-ribosyl)ated PARP1 to linger on DNA thus generating longer PAR polymers. Histones were actually shown to increase the average length of poly(ADP-ribose) generated by PARP1 [34].

**Figure 7:**
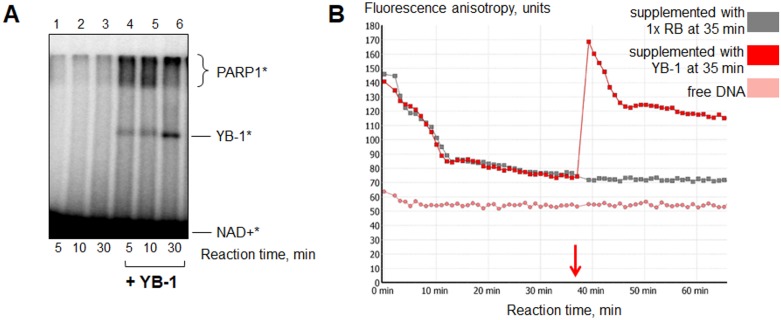
YB-1 can “reactivate” PARylated PARP1 (**A**) PARP1 autopoly(ADP-ribosyl)ation was performed with the use of radioactively labelled NAD+^*^ as described in section *2.5*. The reaction mixtures contained 1x reaction buffer, 10 mM EDTA, 10 nM Nick, 4 μM NAD+^*^ and 200 nM PARP1. After 20 min of reaction, the mixtures were supplemented with 1× reaction buffer (lanes 1–3) or YB-1 to the final concentration of 400 nM (lanes 4–6) and additionally incubated for 5, 10 or 30 min at 37° C. The incubation time after 1× RB/YB-1 addition is indicated at the bottom of the panel. The experiment was performed twice. (**B**) The curves presented illustrate the change of fluorescence anisotropy of FAM-labelled DNA Nick measured by kinetic scanning. The reaction mixtures contained 1× RB, 200 nM PARP1, 10 nM Nick and 10 mM EDTA. Poly(ADP-ribosyl)ation was started by the addition of NAD+ to a final concentration of 500 μM. After 35 min of reaction the samples were supplemented with 1× RB (grey curve) or YB-1 to the final concentration of 400 nM (red curve). All the measurements were carried out in duplicates for each reaction mixture.

YB-1 is positively charged in the reaction conditions used (pH = 8.0, pI(YB-1) = 9.87); moreover, it can interact with PARP1 [31], DNA [47, 48] and poly(ADP-ribose) [31, 49]. In this regard, this protein is a good candidate for stabilization of the catalytically active PARP1-DNA complex. To test this, we performed PARP1 auto-poly(ADP-ribosyl)ation until accumulation of reaction products was ceased due to repulsion of poly(ADP-ribosyl)ated PARP1 from DNA. Then the mixtures were supplemented with 400 nM YB-1 or 1x reaction buffer as a control and additionally incubated at 37° C. We actually found that YB-1 can partially “reactivate” PARP1 automodified in the absence of magnesium (10 mM EDTA) (Figure 7A). This “reactivation” of PARylated PARP1 could also be observed in real-time (Figure 7B). The phenomenon may be accounted for the YB-1-mediated connection of PARP1 molecules, modified by short PAR chains, and the DNA-cofactor. (This case may be described as the formation of hypothetical YB-1-(PAR-PARP1^*^)-DNA complex, in which YB-1 is bound to PAR polymers attached to PARylated PARP1; Supplementary Figure 3 (5)).

Interestingly, according to the literature YB-1 can facilitate assembly of supramolecular structures containing nucleic acids. For example, YB-1 is one of the major proteins of RNA granules [50, 51] and can force alignment of two interacting DNA helices as was shown by AFM [52]. It should be mentioned that YB-1 modification by “reactivated” PARP1 was rather poor (Figure 7A, lanes 4–6). These results indicate that the initial presence of YB-1 in the ternary complex with PARP1 and damaged DNA (hypothetical YB-1-PARP1-DNA complex) is necessary for effective YB-1 poly(ADP-ribosyl)ation. In the present case YB-1 appears to bind poly(ADP-ribose) rather than DNA [31] thus positioning itself relatively far from the active center of PARP1. To test this, we examined if the initial presence of poly(ADP-ribose) in the reaction may disconnect functional interaction of YB-1 and PARP1, resulting in decrease of YB-1 modification.

### YB-1-PARP1 interplay is regulated by poly(ADP-ribose)

**Figure 8:**
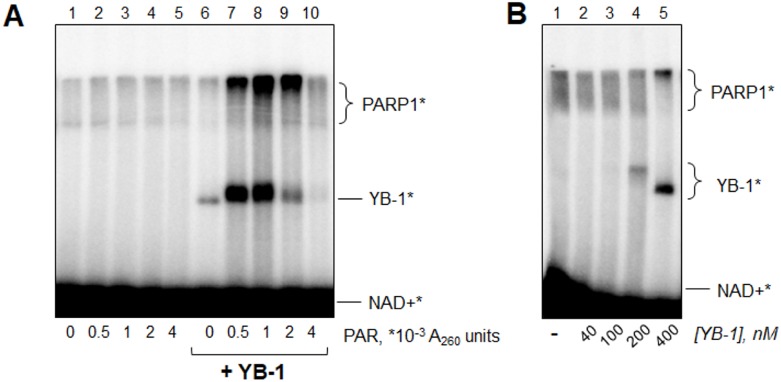
YB-1 and PARP1 interplay is regulated by poly(ADP-ribose) (**A**) Reaction mixtures (10 μl) contained 10 nM Nick, 200 nM PARP1, 0 or 400 nM YB-1, 10 mM EDTA, 4 μM NAD+^*^ and 0-0.004 A_260_ units of PAR prepared according to Materials and Methods, After incubation for 10 min at 37° C, the reaction mixtures were supplemented with 2.5 μl of Laemmli buffer with subsequent heating for 2 min at 97° C and analyzed by SDS-PAGE. The data acquired were analyzed by the Quantity One analysis software, providing the Transform and Crop Plot tools to optimize the image display. The experiment was performed at least 3 times. (**B**) Reaction mixtures (10 μl) contained 10 nM Nick, 200 nM PARP1, 0–400 nM YB-1, 10 mM EDTA and 4 μM NAD+^*^. After incubation for 10 min at 37° C, the reaction mixtures were supplemented by 2.5 μl of Laemmli buffer with subsequent heating for 2 min at 97° C and analyzed by SDS-PAGE. The data acquired were analyzed by Quantity One analysis software, providing the Transform and Crop Plot tools to optimize the image display. The experiment was performed twice.

Total poly(ADP-ribose) free of the DNA-cofactor was prepared according to Materials and Methods and added to the poly(ADP-ribosyl)ation reaction mixture performed in the absence or presence of YB-1 (Figure 8A). It should be mentioned that for this experiment we used a low DNA concentration (10 nM) to observe the full range of YB-1-PARP1 interplay regulation by poly(ADP-ribose). Previously it was shown that YB-1 at a high [YB-1]:[DNA] ratio ([YB-1] >> [DNA]) inhibits PARP1 activity (thus decreasing modification of both PARP1 and YB-1) probably by competing with PARP1 for DNA binding [31]. In the present study, we found that this inhibitory effect of YB-1 disappears during the time of reaction if YB-1 poly(ADP-ribosyl)ation allows this protein to dissociate from the complex with PARP1 and damaged DNA. However, under the conditions used, YB-1 modification is rather weak (Figure 8A, lane 6), indicating PARP1 inhibition by excess of YB-1. As YB-1 affinity for PAR is higher than its affinity for DNA [31], it may be proposed that the initial presence of PAR would lead to attenuation of PARP1 inhibition due to non-covalent binding of excess of YB-1 molecules to poly(ADP-ribose). As a result, PARP1 activity and, particularly, YB-1 PARylation rate should increase.

Actually, we found that samples supplemented with low amounts of poly(ADP-ribose) displayed significantly more pronounced functional interactions of YB-1 and PARP1-modification of YB-1 by PARP1 and YB-1-mediated stimulation of PARP1 activity (increased modification of both PARP1 and YB-1) (Figure 8A, lanes 7 and 8, compare with lane 6 without poly(ADP-ribose)). In contrast, high PAR concentration resulted in total disconnection of functional coupling of YB-1 and PARP1, as was proposed earlier. In this case we observed disappearance of the radioactive band corresponding to modified YB-1 (Figure 8A, lane 10, compare with lane 6).

Interestingly, the presence of moderate PAR amounts (Figure 8A, lane 9) led to decreased YB-1 PARylation rate, while YB-1-mediated stimulation of PARP1 auto-poly(ADP-ribosyl)ation was maintained. This fact speaks in favor the additional mechanisms of PAR influence on poly(ADP-ribosyl)ation other than regulation of stoichiometry of YB-1-PARP1-DNA complexes. Moreover, if PAR-mediated removal of excess YB-1 molecules was the only reason for the increased YB-1-PARP1 functional coupling, we should observe the same effect without PAR addition by reducing the YB-1 concentration. However, this was not the case (Figure 8B). It should be emphasized that PAR itself has no influence on PARP1 activity (Figure 8A, lanes 1–5). So, we conclude that poly(ADP-ribose) and YB-1 can stimulate PARP1 activity when *acting together*.

## DISCUSSION AND CONCLUSIONS

Despite the long story of PARP research (since 1963 [53]), many details of the poly(ADP-ribosyl)ation mechanism are obscure. To date, PARP1 activity was shown to be stimulated by cations [54], polyamines [55] and several basic proteins (histone H1 [34] (pI = 10.84), HMGN1 [35] (pI = 9.6), HMG2L1 [36] (pI = 9.35), and DDB2 [37] (pI = 9.56)). Therefore, the positive charge of the PARP1 effector appears to play an important role, probably by stabilizing the catalytically active PARP1-DNA complex at the initiation and elongation stages. All these proteins were identified as targets of poly(ADP-ribosyl)ation by PARP1, and some of them were also shown to interact with PARP1 or poly(ADP-ribose) [36]. However, the PAR-binding protein XPA was also found to stimulate PARP1 activity [38] in spite of its relatively low isoelectric point (pI = 6.3), suggesting that electrostatic interactions mediated by positively charged effectors may be a prevailing, but subcase of forces maintaining PARP1 in its active conformation.

In the present study, we report new findings concerning functional interactions of PARP1 and the non-canonical DNA repair protein YB-1. Actually, YB-1 has a significant potential for involvement into poly(ADP-ribosyl)ation system due to its high isoelectric point (pI = 9.87) as well as its disordered structure prone to post-translational modifications [56, 57] and allowing YB-1 to interact with DNA [47, 48], poly(ADP-ribose) [31, 49], and PARP1 [31]. We found that YB-1 can form heteromeric complex with PARP1 on DNA damage, serving as preferable PAR acceptor at the initiation stage. During elongation unmodified YB-1 molecules appear to non-selectively bind growing polymers of poly(ADP-ribose) rather than DNA at the PARP1 boarding site (see chapter “Stimulation of PARP1 auto-poly(ADP-ribosyl)ation by YB-1 is partially PAR-mediated” and [31].

We found that YB-1 can significantly stimulate PARP1 activity in conditions of magnesium absence/EDTA presence in a histone-like manner [42]. The C-terminal domain of YB-1 (or its proximal part in the case of YB-1 nuclear form), providing YB-1 multivalence and high net charge, and subsequent ability to bind poly(ADP-ribose), was shown to be indispensable for stimulation of PARP1 activity.

We also discovered that YB-1-PARP1 interplay may be regulated and even mediated by poly(ADP-ribose). Of special interest is PARP1 stimulation by simultaneous addition of poly(ADP-ribose) and YB-1, that was not observed separately for PAR (Figure 8A, lanes 2–4) or YB-1 (Figure 8A, lane 6), as well as couldn't be accounted only for optimization of YB-1 stoichiometry in hypothetical YB-1-PARP1-DNA complexes (Figure 8B). Moreover, in this case YB-1 is bound to free PAR polymers in solution that excludes the variant of PARP1 ”reactivation” discussed in the section “Stimulation of PARP1 auto-poly(ADP-ribosyl)ation by YB-1 is partially PAR-mediated”. It may be proposed that free poly(ADP-ribose) and YB-1 can form multimeric assemblies involving damaged DNA and PARP1 (hypothetical YB-1-PAR-PARP1-DNA complexes, Supplementary Figure 3 (7)), similar to the process of non-membranous compartmentalization driven by PAR and intrinsically disordered proteins in the nucleus [8]. Previously PAR was proposed to act as molecular glue, enhancing protein residence times and interactions [5]. According to this supposition, PARP1 activity may be increased by limitation of PARP1 dissociation from the catalytically active complex with DNA or by a rise in its (or DNA) effective concentration within the compartment.

In the present study, most experiments on the functional interactions of YB-1 and PARP1 were performed at a [YB-1] : [PARP1] molar ratio ~ 2 : 1 or 4 : 1 (400 nM YB-1 and 100–200 nM PARP1). According to the literature, eukaryotic cells contain approximately 2 × 10^5^ PARP1 molecules per nucleus [58, 59]. The YB-1 protein level has been estimated as 5 × 10^5^ YB-1 molecules per cell in human cancer cells (by calibration of YFP fluorescence to protein molecules) [60] and as 20 × 10^5^ YB-1 molecules per cell in mouse fibroblasts (by mass spectrometry data) [59]. Unlike what occurs with PARP1 [61], YB-1 is distributed between the nucleus and the cytoplasm, complicating the quantitative estimation of YB-1 nuclear concentration. However, a lot of data speaks in favor of nuclear relocalization of YB-1 induced by genotoxic stress [62–64]. Moreover, as mentioned earlier, YB-1 nuclear localization is a feature of tumor cells [16], especially those resistant to chemotherapy [17]. Cohen and co-authors also demonstrated that in human cancer cells YB-1 accumulates in the nucleus [60]. According to the results of western blot analysis obtained by Koike and co-authors, there is about 10 times more YB-1 in the cytosol than in the nuclear fraction [62]. However, the same research revealed significantly increased amounts of YB-1 in the nucleus after UV irradiation (up to 50% as can be seen by Western-blot) [62]. Based on the facts listed above, we can speculate that under DNA-damaging stress YB-1 nuclear concentration would exceed 2.5–10 × 10^5^ molecules per nucleus. Thus, the effects investigated in this study could be applied *in vivo*.

In spite of alterations in PARP1 expression levels and its enzymatic activity in cancer cells have been intensively studied recent years, there is no consensus among researchers regarding either decreased or increased PARP1 activity may result in tumor resistance to PARPi. Specifically, by insertional mutagenesis screen, it was found that *PARP1* loss-of-function mutants were 100-fold more resistant to olaparib than cells with a normal genetic background [65]. Moreover, Liu and co-authors reported that cells resistant to treatment with temozolomide and PARPi ABT-888 had decreased PARP1 expression levels [66]. It was also shown that cancer cells with undetectable endogenous PAR tend to show greater resistance to PARPi, as compared with cells generating PAR at detectable levels [67].

It is well known that tumors in patients carrying hereditary mutations in the HR genes generally display increased sensitivity to PARPi and other DNA-damaging treatment such as platinum-based chemotherapy [68]. In this case, secondary mutations restoring the wild-type *BRCA2* reading frame may result in acquired resistance of *BRCA2*-mutated cancer cells both to cisplatin and to PARPi [69]. Based on the finding that PARP1 is hyperactivated in HR-defective tumors, while PARP inhibitor-resistant *BRCA2*-mutant cells revert back to normal levels of PARP activity [70], Gottipati and co-authors also conclude that increased PARP1 activity correlates with an increased sensitivity to PARPi [70].

However, the possibility that decreased PARP1 activity in tumor cells may result in resistance to PARPi has not yet been validated clinically [15]. Another hypothesis, by contrast, is that PARP1 activity is increased due to its overexpression in tumors [61, 71] or PARP1 phosphorylation at Tyr907 by the tyrosine kinase c-Met [72, 73]. Specifically, in accordance with this supposition, resistance to olaparib treatment was observed by Gilabert and co-authors as a feature of breast cancer stem cells with the highest level of PARP1 overexpression [74]. To explain this result, the authors hypothesized that PARP1 overexpression could improve DNA repair capacity of the cells thus promoting resistance to DNA-damaging treatment, including olaparib [74]. It was also proposed that in such cases overexpression of the target may require higher drug concentration for effective inhibition [74].

PARP1 upregulation and its increased activation accompanied by elevated levels of PAR-containing proteins were actually observed in cisplatin-resistant cell clones [71, 75]. It should be emphasized that increased PARP1 activity in cisplatin-resistant cells was revealed in the absence of obvious HR defects [10], and olaparib was reported to sensitize tumors to this DNA damaging drug [75]. Interestingly, YB-1^+/−^ cells were shown to have increased sensitivity to cisplatin compared to YB-1^+/+^ cells [24]. Based on the data obtained, we can propose that increased PARP1 activation in cisplatin-resistant cells may be due to its stimulation by oncoprotein YB-1. According to our results, YB-1 can interfere with the action of PARP1 inhibitors taken in concentrations insufficient to completely inhibit PARylation process (0–87.5 μM 3-aminobenzamide, Figure 6A; 0–150 nM olaparib, Figure 6B; 0–125 mg/l EtBr, Figure 6C), but indeed is unable to stimulate PARP1 activity in the presence of high concentrations of PARP1 inhibitors, including olaparib (≥200 nM olaparib, Figure 6B).

**Figure 9:**
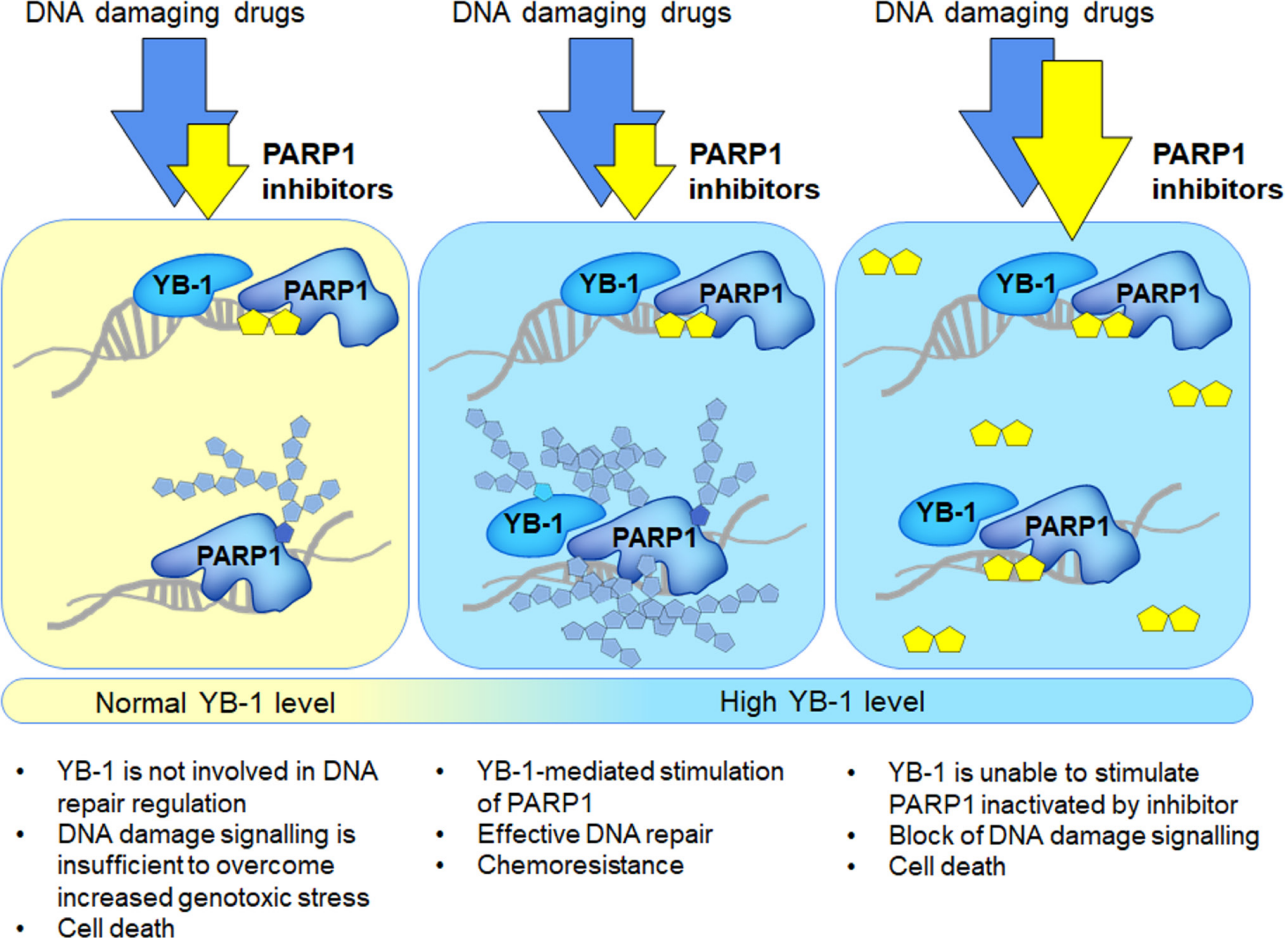
Application of PARP1 inhibitors to the treatment of YB-1-overexpressing tumors (scheme)

To conclude, the present study provides a possible mechanism of chemoresistance mediated by the oncoprotein YB-1. We can speculate that application of PARP1 inhibitors in addition to DNA damaging agents during anticancer treatment may be specifically beneficial in the case of tumors overexpressing YB-1. However, due to YB-1-mediated increased activation of PARP1, it should be taken into account that the therapy of tumors overexpressing YB-1 may require elevated effective doses of PARP1 inhibitors (Figure 9).

## MATERIALS AND METHODS

### Proteins and reagents

Recombinant histidine-tagged analogs of YB-1 protein and its nuclear form YB-1(1-219) were produced by expression in *Escherichia coli* BL21(DE3) and purified as described previously [76]. Recombinant PARP1 was purified as described earlier with minor modifications [77]. The plasmid pET-3-1-YB-1 containing the human YB-1 cDNA was generous gift from Lev P. Ovchinnikov and Dmitry Kretov (Institute of Protein Research RAS, Moscow, Russia). The plasmid DNA containing the human PARP1 cDNA was a kind gift of Dr. M. Satoh (Université Laval, Québec, Canada).

Yeast nicotinamide mononucleotide adenylyltransferase (NMAT) and phage T4 polynucleotide kinase were kindly provided by Dr. Stanislav I. Shram (IMG RAS, Moscow, Russia) and Dr. Irina O. Petruseva (ICBFM SB RAS, Novosibirsk, Russia), respectively. The AP-CSD YB-1 mutant was a kind gift from Lev P. Ovchinnikov and Dmitry Kretov. NAD+ and β-nicotinamide mononucleotide were from Sigma (USA), [α-^32^P]ATP and [γ-^32^P]ATP were from ICBFM SB RAS. Olaparib was from Selleckchem.

### Oligonucleotides

Oligonucleotides (Table 1) were from Biosset (Novosibirsk, Russia). To obtain the DNA duplex Nick, oligonucleotide ODN1 was annealed at a 1:1.2 molar ratio to the corresponding complementary strands ODN2 and ODN3. For gel-shift experiments ODN1 before annealing was 5′-radioactively labeled using [γ-^32^P]ATP with T4 polynucleotide kinase.

**Table 1:** Oligonucleotide sequences and designations

ODN1	5′-ggaag accct gacgt ttccc aactt tatcg ccF-3′
ODN2	5′-ggcga taaag ttggg-3′
ODN3	5′-aa acgtc agggt cttcc-3′
Nick (ODN1 + ODN2 + ODN3)	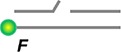

Letter F in the nucleotide sequence and on the scheme designates 5(6)-carboxyfluorescein (FAM) residues.

Designation and schematic representation of the DNA duplex used in the study.

### NAD+^*^ synthesis

The reaction mixture (50 μl) containing 2 mM β-nicotinamide mononucleotide, 1 mM ATP, 0.5 mCi of [α-^32^P]ATP, 5 mg/ml NMAT, 25 mM Tris-HCl (pH 7.5) and 20 mM MgCl_2_ was incubated for 1 h at 37° C. The enzyme was then denatured at 97° C for 3 min and precipitated by centrifugation.

### Protein separation and analysis

Reaction mixtures were analyzed by denaturing polyacrylamide gel electrophoresis according to Laemmli [78] with modifications. Briefly, a step gradient separating gel of 4% and 10% (70:1 acrylamide:bisacrylamide ratio, pH 8.8) was used. Samples were supplemented with Laemmli loading buffer and heated for 1.5 min at 97° C. The positions of protein bands were visualized by phosphor-imaging with Typhoon FLA 7000 (GE Healthcare). Alternatively, the samples were applied onto chromatography paper (GE Healthcare) saturated with 5% TCA. TCA-targets were purified from free [^32^P]-radioactively labelled NAD+ (NAD+^*^) by washing 1 × 10 min in 10% TCA, 3 × 5 min in 5% TCA and 1 × 10 min in EtOH. The total radioactivity of the reaction pro ducts was compared by phosphorimaging with Typhoon FLA 7000 (GE Healthcare).

### Poly(ADP-ribosyl)ation by PARP1

The reaction mixture (total volume 10 μl) contained 1x reaction buffer (50 mM Tris-HCl, pH 8.0, 40 mM NaCl, 1 mM DTT), 5 mM MgCl_2_/10 mM EDTA, 0.2 μM PARP1, 0–3200 nM YB-1 (or its mutants), 10–100 nM Nick and PARP1 inhibitor (0–700 μM 3-aminobenzamide, 0–500 nM olaparib or 0–50 mg/l EtBr) or 0–0.004 A_260_ units/ml of poly(ADP-ribose) if necessary. Blending of the reaction components was performed on ice. The reaction was initiated by addition of NAD+^*^ to a final concentration of 4–500 μM. The mixtures were incubated at 37° C for 0–30 min. The reactions were stopped by heating at 97° C for 1.5 min or by applying the samples onto TCA-targets to inhibit PARP1 activity. Samples were analyzed as described previously (section “Protein separation and analysis”). Experiments were performed at least 3 times.

### Real-time assay for poly(ADP-ribosyl)ation

PARP1 and YB-1 binding to damaged DNA Nick and their dissociation from the damage site during the poly(ADP-ribosyl)ation process was detected by the fluorescent spectroscopy technique [79] (Figure 3). The samples (10 μl) were prepared on ice according to (Materials and Methods “Poly(ADP-ribosyl)ation by PARP1”) in Corning black 384-well polystyrene assay plates and incubated for 5 min at room temperature. Fluorescence anisotropy of FAM-labelled DNA Nick was measured by kinetic scanning at 25° C with the use of a CLARIOstar multifunctional microplate reader (BMG LABTECH GmbH, Germany). The fluorescent probes were excited at 495 nm and the fluorescence intensity was detected at the emission maximum (520 nm).

To analyze YB-1 binding to DNA or to the PARP1-DNA complex, fluorescent anisotropy values were measured before NAD+ addition. The data were plotted (A against C) and fitted by 4-parameter logistic equation: *A = A_0_ + (A_∞_ – A_0_)/[1 + (EC_50_/C)^n^]* , where A is the measured fluorescence anisotropy of a solution containing the FAM-labelled DNA at a given concentration (C) of YB-1 and 0 or 200 nM PARP1. A_0_ is the fluorescence anisotropy of FAM-labelled DNA alone (or in the presence of 200 nM PARP1). A_∞_ is the fluorescence anisotropy of the labelled DNA saturated with the partner proteins. EC_50_ is the concentration of YB-1 at which A − A_0_ = (A_∞_ − A_0_)/2. n is the Hill coefficient, which denotes the steepness (slope) of the nonlinear curve. To analyze PARP1 and YB-1 dissociation from the complexes with damaged DNA during poly(ADP-ribosyl)ation, the reaction mixtures were supplemented with NAD+ to a final concentration of 500 μM. The data were analyzed by MARS Data Analysis Software (BMG LABTECH GmbH, Germany). All the measurements were carried out in duplicates for each specific condition and performed at least 3 times.

### Gel-mobility shift analysis

To study YB-1-PARP1-DNA complex formation, the reaction mixtures (10 μl) contained 1x reaction buffer, 40 nM radioactively labelled Nick, 0 or 200 nM PARP1 and YB-1 at various concentrations. After 10 min of incubation at 37° C, the samples were supplemented with 2.5 μl of loading buffer (reaction buffer RB, 20% glycerol, 0.025% bromophenol blue).

To study DNA release during poly(ADP-ribosyl)ation, the reaction was performed according to (Materials and Methods, “Poly(ADP-ribosyl)ation by PARP1”) with the use of radioactively labelled Nick for PARP1 activation. Aliquots were removed between 0–60 min of incubation and supplemented with 1 μM olaparib to inactivate PARP1 and 2.5 μl of loading buffer (reaction buffer RB, 20% glycerol, 0.025% bromophenol blue).

The samples were chilled on ice and loaded onto a cooled and pre-equilibrated 7.6% polyacrylamide native gel (76:1 acrylamide:bisacrylamide, 25 mM Tris-borate buffer, pH 8.3). Electrophoresis was conducted at 10 V/cm at 4° C by using 25 mM Tris-borate buffer, pH 8.3 as electrode buffer. The gels were dried, and the positions of YB-1-DNA complexes were visualized by phosphorimaging with by Typhoon FLA 7000 (GE Healthcare).

### Preparation of total poly(ADP-ribose)

Total poly(ADP-ribose) was obtained as described previously [31] with minor modifications. Briefly, DNA was removed by benzonase treatment and PAR was isolated from the resulting sample by phenol:chloroform:isoamyl alcohol (25:24:1) extraction. PAR was additionally purified by ethanol precipitation and dissolved in the reaction buffer to the final concentration of 1 A_260_ units/ml.

### Statistical analysis of the data

All experiments were performed at least three times. The quantitative data were analyzed using MS Excel 2010 and presented on histograms as the Mean ± SD.

## SUPPLEMENTARY MATERIALS AND FIGURES



## Abbreviations

YB-1: Y-box binding protein 1
PARP1: poly(ADP-ribose) polymerase 1
PAR: poly(ADP-ribose)
