# Supplementary material for: The multifunctional protein YB-1 potentiates PARP1 activity and decreases the efficiency of PARP1 inhibitors
Source: Oncotarget. 2018 May 4;9(34):23349–65. doi: 10.18632/oncotarget.25158 (PMC5955111; doi:10.18632/oncotarget.25158)
Supplement: Supplementary file 1 [file oncotarget-09-23349-s001.pdf]

The multifunctional protein YB-1 potentiates PARP1 activity and decreases the efficiency of PARP1 inhibitors

SUPPLEMENTARY MATERIALS

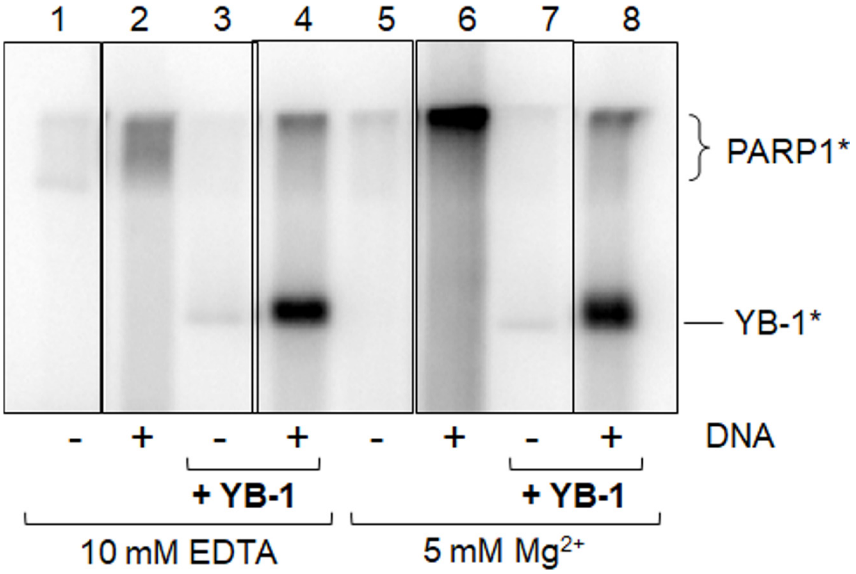

Supplementary Figure 1: YB-1 is unable to stimulate PARP1 activity in the absence of damaged DNA. Reaction mixtures (10 µl) contained 1× RB, 10 mM EDTA or 5 mM Mg^{2+}, 4 µM NAD+*, 200 nM PARP1, 0 or 100 nM Nick and 0 or 400 nM YB-1. After incubation for 10 min at 37° C, the reaction mixtures were supplemented with 2.5 µl of Laemmli buffer with subsequent heating for 2 min at 97° C and analyzed by SDS-PAGE. Poly(ADP-ribosyl)ation was performed in the presence: lane 1 – EDTA; lane 2 – EDTA, Nick; lane 3 – EDTA, YB-1; lane 4 – EDTA, Nick, YB-1; lane 5 – Mg^{2+}; lane 6 – Mg^{2+}, Nick; lane 7 – Mg^{2+}, YB-1; lane 8 – Mg^{2+}, Nick, YB-1. The data acquired were analyzed by the Quantity One analysis software, providing the Transform and Crop Plot tools to optimize the image display. The experiment was performed at least 3 times.

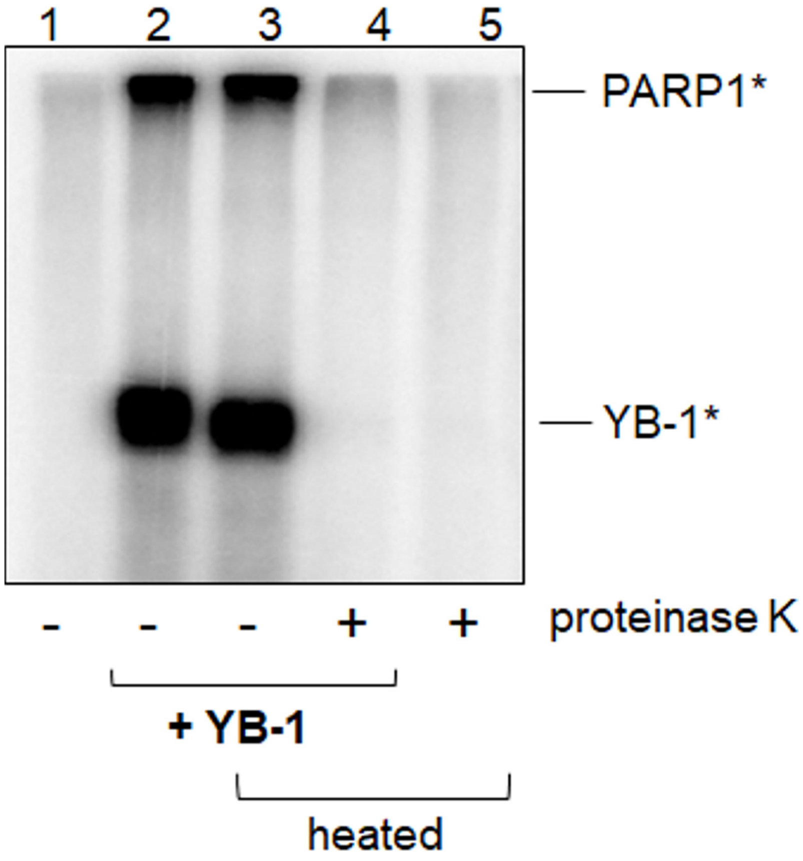

Supplementary Figure 2: YB-1 degraded by proteinase K treatment is unable to stimulate the activity of PARP1 (control). Reaction mixtures (10 µl) contained 1x RB, 10 mM EDTA, 4 µM NAD+*, 100 nM PARP1, 100 nM Nick, 0 or 400 nM YB-1, and 0 or 1 µg of proteinase K inactivated by heating for 10 min at 97° C. After incubation for 10 min at 37° C, the reaction mixtures were supplemented with 2.5 µl of Laemmli buffer with subsequent heating for 2 min at 97° C and analyzed by SDS-PAGE. Lane 1 – PARP1 activity. Lane 2 – the activity of PARP1 in the presence of 400 nM YB-1. Lane 3 – the activity of PARP1 in the presence of 400 nM YB-1, preliminary heated for 10 min at 97° C. Lane 4 – the activity of PARP1 in the presence of 400 nM YB-1, preliminary incubated with proteinase K (20 min at 37° C) and subsequently heated for 10 min at 97° C. Lane 5 – the activity of PARP1 in the presence of proteinase K, preliminary heated for 10 min at 97° C. The data acquired were analyzed by the Quantity One analysis software, providing the Transform and Crop Plot tools to optimize the image display.

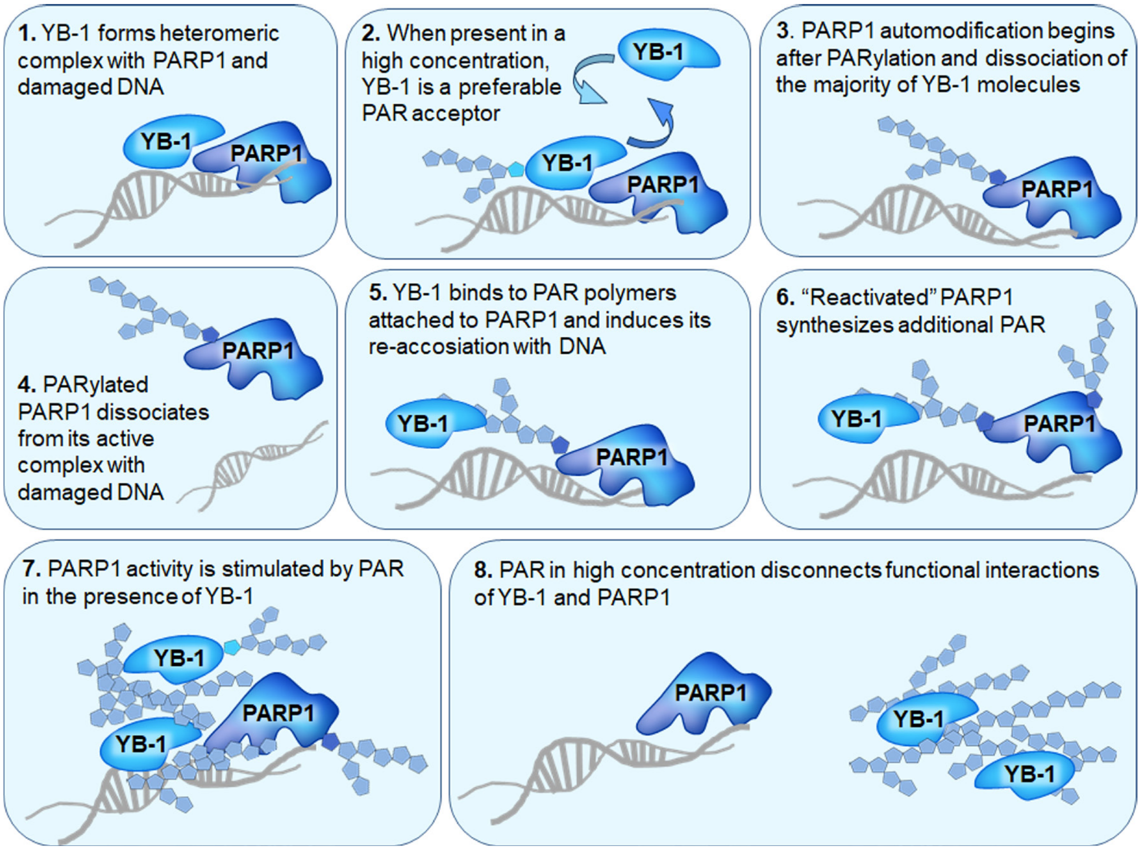

Supplementary Figure 3: YB-1-dependent mechanisms of regulation of PARP1 activity (scheme).
